# HSPA4 upregulation induces immune evasion via ALKBH5/CD58 axis in gastric cancer

**DOI:** 10.1186/s13046-024-03029-4

**Published:** 2024-04-08

**Authors:** Daqin Suo, Xiaoling Gao, Qingyun Chen, Tingting Zeng, Jiarong Zhan, Guanghui Li, Yinli Zheng, Senlin Zhu, Jingping Yun, Xin-Yuan Guan, Yan Li

**Affiliations:** 1 State Key Laboratory of Oncology in South China, Guangdong Provincial Clinical Research Center for Cancer, Sun Yat-sen University Cancer Center, Guangzhou, 510060 China; 2 The clinical Laboratory Center, Hainan General Hospital, Hainan affiliated Hospital of Hainan Medical University, Haikou, 570311 China; 3 Medical Research Institute, Guangdong Provincial People’s Hospital (Guangdong Academy of Medical Sciences), Southern Medical University, Guangzhou, 510080, China; 4 The First Affiliated Hospital, Sun Yat-sen University, Guangzhou, 510080 China; 5 Department of Clinical Oncology, The University of Hongkong, Hong Kong, China

**Keywords:** HSPA4, ALKBH5, CD58, Immunotherapy, Gastric cancer

## Abstract

**Introduction:**

Gastric cancer (GC) is one of the leading causes of cancer-related death worldwide. Recently, targeted therapies including PD1 (programmed cell death 1) antibodies have been used in advanced GC patients. However, identifying new biomarker for immunotherapy is still urgently needed. The objective of this study is to unveil the immune evasion mechanism of GC cells and identify new biomarkers for immune checkpoint blockade therapy in patients with GC.

**Methods:**

Coimmunoprecipitation and meRIP were performed to investigate the mechanism of immune evasion of GC cells. Cocuture system was established to evaluate the cytotoxicity of cocultured CD8^+^ T cells. The clinical significance of HSPA4 upregulation was analyzed by multiplex fluorescent immunohistochemistry staining in GC tumor tissues.

**Results:**

Histone acetylation causes HSPA4 upregulation in GC tumor tissues. HSPA4 upregulation increases the protein stability of m^6^A demethylase ALKBH5. ALKBH5 decreases CD58 in GC cells through m^6^A methylation regulation. The cytotoxicity of CD8^+^ T cells are impaired and PD1/PDL1 axis is activated when CD8^+^ T cells are cocultured with HSPA4 overexpressed GC cells. HSPA4 upregulation is associated with worse 5-year overall survival of GC patients receiving only surgery. It is an independent prognosis factor for worse survival of GC patients. In GC patients receiving the combined chemotherapy with anti-PD1 immunotherapy, HSPA4 upregulation is observed in responders compared with non-responders.

**Conclusion:**

HSPA4 upregulation causes the decrease of CD58 in GC cells via HSPA4/ALKBH5/CD58 axis, followed by PD1/PDL1 activation and impairment of CD8^+^ T cell’s cytotoxicity, finally induces immune evasion of GC cells. HSPA4 upregulation is associated with worse overall survival of GC patients with only surgery. Meanwhile, HSPA4 upregulation predicts for better response in GC patients receiving the combined immunotherapy.

**Supplementary Information:**

The online version contains supplementary material available at 10.1186/s13046-024-03029-4.

## Introduction

Gastric cancer (GC) is the fifth most common neoplasm and the fourth deadly cancer worldwide [1]. It is the second most commonly diagnosed cancer and the third leading cause of cancer-related deaths in China [2]. Over 95% of gastric cancers are adenocarcinomas, which are typically classified based on anatomic location and histologic type[3]. Patients with GC generally carry a poor prognosis because it is often diagnosed at an advanced stage [3].

Recently, targeted therapies including PD1 (programmed cell death 1) antibodies, such as pembrolizumab and nivolumab, have been approved by the Food and Drug Administration for use in advanced GC patients [3]. Targeted therapies have produced encouraging results in clinical trials for the treatment of patients with locally advanced or metastatic GC disease [4]. The implementation of biomarker testing, especially the expression of PDL1 (programmed cell death 1 ligand 1) and microsatellite instability (MSI) status, has had a significant impact on clinical practice. Although several studies demonstrated that the expression of PDL1 could predict the clinical response to anti-PDL1 treatment in multi-tumors [5–7], some clinical trials indicated that PDL1 combined positive score (CPS) had a poor power to predict the overall survival and objective response rate (ORR) between nivolumab plus chemotherapy treatment groups and chemotherapy alone treatment groups in advanced gastric/gastro-oesophageal junction/oesophageal adenocarcinoma [4, 8]. MSI-H (high-frequency microsatellite instability) is considered as a biomarker for pembrolizumab therapy among patients with advanced gastric/esophageal junction cancer [9]. However, there are some different reports, for example, the survival benefits obtained from the combination of nivolumab and chemotherapy are not related to MSI status [4]. Therefore, identifying new biomarker for anti-PD1 immunotherapy is still urgently needed.

Heat shock proteins (HSPs) are a highly conserved family of molecular chaperones, which are induced in a challenging environmental or pathological stress [10]. HSPs participate in protein assembly, secretion, transport, protein degradation, and transcription factor regulation, thereby maintaining protein homeostasis [11]. They are also involved in many biological processes in cancer cells, such as regulation of cell proliferation, angiogenesis and evasion of apoptosis [12–14]. Aberrant expression of HSPs was reported in a wide range of cancers, including lung cancer, breast cancer, prostate cancer and ovarian cancer, and was associated with adverse prognosis [15–18]. Potential of targeting HSPs was explored and inhibitors of HSPs showed anti-cancer effects. For example, VER-155,008 is an HSPs inhibitor that promotes tumor cells apoptosis by reducing the expression of Hsp70 and Hsp90 [19]. Pinaverium bromide, a spasmolytic agent, inhibits the intracellular chaperone activity of Hsp70 system and elicits cytotoxicity in melanoma cells [20]. Debio-0932 is a second generation Hsp90 inhibitor with high affinity binding to Hsp90 [21]. A clinical trial exhibited that Debio-0932 had limited clinical activity and further development as adjunct treatment of NSCLC was warranted [21]. These studies suggest that HSPs play an essential role in tumor progression and might be potential therapeutic target.

HSPA4 (heat shock protein family A (Hsp70) member 4), a representative of the Hsp110 family, is upregulated in various cancer types [20, 22, 23]. HSPA4 upregulation is correlated with poor overall survival (OS) in HNSC and hepatocellular carcinoma (HCC) [22, 24]. Knockdown of HSPA4 retarded the progression and development of colorectal cancer cells [25]. Accumulating evidences showed that HSPA4 might not only participate in the progression but also immune regulation in some cancers. Bioinformatics analysis suggested that HSPA4 upregulation was positively related to immune cell infiltration and immune checkpoints (PD1 and CTLA-4) in HCC [22]. In addition, B cells selectively produced pathogenic IgG antibodies targeting glycosylated membrane protein HSPA4 and promoted lymph node metastasis in breast cancer [26].

In this study, we illustrate that HSPA4 is upregulated in GC tumor tissues and mediates immune escape of tumor cells. We further uncover the molecular mechanism by which HSPA4/ALKBH5/CD58 axis upregulates PDL1 expression in gastric cancer cells and inhibits the cytotoxicity of CD8^+^ T cells in tumor environment. In addition, we report that, although HSPA4 upregulation is correlated with poor prognosis in patients with GC, HSPA4 upregulation could be a valuable biomarker for predicting better response to PD1 checkpoint blockade therapy for GC patients.

## Materials and methods

### Patients and specimens

Thirty-seven pairs of GC tumor and non-tumor tissues were collected at Sun Yat-Sen University Cancer Center (SYSUCC) (Guangzhou, China). These tissues were used for qPCR and western blotting analyses. A total of 94 pairs of GC tumor and non-tumor tissues were collected at The First Affiliated Hospital, Sun Yat-sen University (SYSUFAH) (Guangzhou, China). These patients did not receive any treatment except for surgery. The study was approved by the Committees for Ethical Review of Research Involving Human Subjects at SYSUCC and SYSUFAH. This study was conducted in accordance with ethical guidelines of the Declaration of Helsinki.

A total of 40 tumor tissues were collected from GC patients receiving anti-PD1 immunotherapy combined with chemotherapy (SYSUCC). Thirty-two patients received post-surgery treatment and 8 received both before- and post-surgery treatment. The main chemotherapy regimen was FOLFOX (Fluorouracil + Oxaliplatin) or XELOX (Capecitabine + Oxaliplatin). Patients were divided into two groups according to the therapy effect: responders (R) and non-responders (NR). Responders were identified as the patients experiencing a confirmed complete response (CR) or partial response (PR). Non-responders were identified as the patients experiencing a confirmed progressive disease (PD) or stable disease (SD). This study was approved by the Committees for Ethical Review of Research Involving Human Subjects at SYSUCC. This study was conducted in accordance with ethical guidelines of the Declaration of Helsinki.

### Cell lines

Human GC cell lines AGS, MKN45 and HGC27 were obtained from American type culture collection (ATCC, MD) and cultured in RPMI1640 (Gibco BRL, NY) supplemented with 10% fetal bovine serum (ExCell Bio, Shanghai, China) and 1% penicillin/streptomycin (Invitrogen, NY, USA). All cells were incubated with 5% CO_2_ at 37 °C in a humidified incubator. The cell lines were validated by STR profiling.

### Animal studies

All animal experiments were approved by the Animal Ethics Committee at Sun Yat-sen University Cancer Center (SYSUCC). BALB/c nude mice (female, 4-weeks old) were purchased from the Beijing Vital River Laboratory Animal Technology (Beijing, China). Animals were housed under specific pathogen-free conditions. To evaluate the xenograft growth, 1 × 10^6^ HSPA4-overexpressing cells (MKN45-HSPA4) or control cells (MKN45-Vec) suspended in 100 µl phosphate buffered saline (PBS) were injected into the flanks of nude mice (*n* = 5). The length (L) and width (W) of the tumor were measured every 5 days. Tumor volumes were calculated as volume (mm^3^) = L×W^2^ × 0.5. Tumor tissues (50 mg per tumor) were frozen rapidly in liquid nitrogen, and ground using tissue grinder. RIPA lysis buffer was used to lyse the tissues on ice for 30 min. The supernatant was collected after centrifuging at 12,000 g for 20 min at 4 °C. Western blotting analysis was performed to analyze the expression of HSPA4 in xenografts.

### Plasmids and siRNAs

pEZ-Lv105-HSPA4, psi-LVRU6GP-shRNAs targeting HSPA4 and the corresponding control plasmids were purchased from GeneCopoeia (Guangzhou, China). Lentivirus was packaged using the Lenti-Pac™ HIV expression Packaging Kit (GeneCopoeia, Guangzhou, China) in 293FT cells (Invitrogen, NY, USA). siRNAs targeting ALKBH5 were obtained from RiboBio (Guangzhou, China). siRNAs were transfected into cells using Lipofectamine 3000 (Invitrogen, NY, USA) according to the manufacturer’s instructions.

### Cell proliferation assay

Cells were plated at a density of 500 (AGS) or 1000 (HGC27 and MKN45) cells per well in 96-well plates. After 24 h, cell viability was detected by Cell Counting Kit-8 (CCK-8; Dojindo, Kumamoto, Japan) according to the manufacturer’s instructions. OD450 was examined once a day for five days. Three independent assays were repeated.

### Foci formation assay

A total of 500 cells (AGS) per well were seeded into 6-well plates and cultured for 12 days. Cell colonies (> 50 cells/colony) were fixed with 75% ethanol, stained with 1% crystal violet and counted. Three independent assays were repeated.

### 5-ethynyl-2’-deoxyuridine (EdU) assay

EdU assay were performed using Cell-Light 5-ethynyl-2’-deoxyuridine (EdU) Apollo488 in vitro Kit (Ribobio, Guangzhou, China). Briefly, GC cells were plated in 6-well plates, and incubated with 1× EdU assay buffer for 2 h. After washing, the cells were incubated with Apollo®488 staining for 30 min. The EdU(+) cells were analyzed by flow cytometry.

### Quantitative real time PCR (qRT-PCR)

Total RNA was extracted from cells using TRIzol Reagent (Invitrogen, NY) and subjected to RT-PCR using gene-specific primers. SYBR Green Master Rox (Vazyme, Nanjing, China) was used for qRT-PCR analysis. The primers’ sequences were listed in Supplementary materials.

### Cycloheximide chase assay and western blotting

Cells were treated with 10 µM cycloheximide (CHX) (Santa Cruz Biotechnology, Santa Cruz, CA) and protein lysates were harvested at different time points. Western blotting analysis was performed according to the standard protocol. Chemiluminescence signals were captured and analyzed by Quantity One system (Bio-Rad, Hercules, CA, USA). Antibodies are listed in the Supplementary materials.

### Immunofluorescence (IF) staining

Paraffin-embedded formalin fixed sections were de-paraffinized with xylene, rehydrated with graded ethanol, then incubated with 3% hydrogen peroxide for 10 min at 37 °C. Antigen retrieval was performed in EDTA buffer (pH 8.0) for 8 min. The slides were blocked with 4% bovine serum albumin for 30 min at room temperature, then slides were incubated with anti-CD58 (1:50), anti-ALKBH5 (1:2000) at 4 °C overnight in a moist chamber. After thorough washing, the slides were incubated with Alexa Fluor 633– or 488–conjugated secondary antibodies (Invitrogen, NY). Subsequently, all slides were mounted with anti-fade reagent 4′,6 diamidino-2-phenylindole (DAPI, Thermo Fisher Scientific, MA). Images were captured with an OLYMPUS FV2000 fluorescence microscope.

### Dot blot assay

Total RNA was spotted onto a Hybond-N + membrane (Byotime, Shanghai, China) and cross-linked using UV CROSSLINKERS (Giangarlo Scientific, NY). The membrane was incubated in blocking buffer (5% skim milk) for 1 h at room temperature and then with an anti-m^6^A antibody overnight at 4 °C. After washing, the membrane was incubated with an anti-mouse antibody for 1 h at room temperature. Finally, the membrane was incubated with ECL luminescent solution, and chemiluminescence signals were captured and analyzed by Quantity One system (Bio-Rad, Hercules, CA, USA). Methylene blue (MB) was used to interact with RNA and as a loading control.

### Coimmunoprecipitation (co-IP) assay

Co-IP assays were performed using a Pierce Direct Magnetic IP/Co-IP kit (Thermo Scientific, Rockford, IL) according to the manual. Briefly, cells were lysed with IP lysis buffer. 500 µl lysis solution at a concentration of 1.5 mg/ml was incubated with antibody-conjugated magnetic beads overnight at 4 °C. The pull-down samples were subjected to immunoblotting assay.

### Methylated RNA immunoprecipitation (Me-RIP)

Me-RIP assays were performed using a riboMeRIP m^6^A Transcriptome Profiling Kit (RiboBio, Guangzhou, China). Briefly, 18 µg total RNA was interrupted by RNA fragmentation buffer. Then, N6-methyladenosine (m^6^A) methylated RNAs were immunoprecipitated with 5 µg anti-m^6^A antibody. After immunoprecipitation, washing and elution, the eluted m^6^A RNA fragments were purified and recycled with Monarch® RNA Cleanup Kit (New England Biolabs, Ipswich, MA). Reverse transcription was performed using the Evo M-MLV RT kit with gDNA Clean for qPCR (Accurate Biology, Changsha, China). Primers were designed according to the m^6^A modification sites predicted by SRAMP (http://www.cuilab.cn/sramp). QRT-PCR was performed using SYBR Green SuperMix (Vazyme, Nanjing, China) and Roche 480 Real-Time PCR system (Roche, Basel, Switzerland).

### CD8^+^ T cells culture

Peripheral blood mononuclear cells (PBMCs) from healthy donors were collected from buffy coats by Ficoll gradient centrifugation. CD8^+^ T cells were isolated from PBMCs using CD8^+^ microbeads (Miltenyi Biotec, North Rhine-Westphalia, Germany) and cultured in complete RPMI medium (RPMI1640, 10% FBS, 10 mmol/L HEPES, 1 mmol/L sodium pyruvate, 2 mmol/L L-glutamine, 0.05 mmol/L 2-mercaptoethanol, and penicillin/streptomycin) (Invitrogen, NY, USA).

### In vitro T cells activation and FACS analysis

CD8^+^ T cells were seeded in CD3 antibody (2 µg/ml) coated plates, and stimulated with CD28 antibody (2 µg/ml) and IL-2 (10 ng/ml) for 24 h. For co-culture experiments, tumor cells (2 × 10^4^ per well for HGC27 and MKN45; 1 × 10^4^ per well for AGS) were plated in 96-well plates. Twelve hours later, 1 × 10^4^ stimulated CD8^+^ T cells were added to the tumor cells in complete RPMI1640 medium with 10 ng/ml IFN-γ. The Plates were centrifuged at 400 g for 5 min to ensure cell-to-cell contact and the co-cultures were kept for 72 h at 37 °C and 5% CO_2_ in a humidified incubator. Then CD8^+^ T cells and tumor cells were analyzed with flow cytometry respectively.

To evaluate intracellular expression of IFN-ɤ, GZMB, and TNF-α of CD8^+^ T cells, CD8^+^ T cells were co-cultured with tumor cells for 16 h, brefeldin A (BFA, BioLegend, San Diego, CA) was added 6 h before cells were analyzed.

### Bioinformatic analysis of RNA-seq and single cell RNA-seq data of GC samples

The gene expression data of GC tumor tissues and normal gastric tissues were obtained from GEPIA (http://gepia.cancer-pku.cn/) that based on samples from TCGA and GTEx databases [27] or Gene Expression Omnibus (GEO) datasets (GSE54129) [28], which have been normalized using the locally weighted scatter plot smoothing (Lowess). The LinkedOmics [29] was analyzed for genes that correlated negatively with ALKBH5 based on the TCGA program in GC tissues. The correlation between ALKBH5 and CD58 was obtained from LinkedOmics based on GC datasets of TCGA and GEO (GSE13861) [29, 30]. The correlation between HSPA4 and tumor proliferation signature was analyzed by R GSVA package, the parameter method=’ssgsea’ was selected, and finally the correlation was analyzed by the Spearman’s correlation. To identify the correlation between CD58 and immune score, RNA-seq profiles were downloaded from TCGA GC dataset, and the immune score was download from TIMER2.0 [31]. The correlation between CD58 and CD8^+^ T cells immune score was drawn by R ggstatsplot package.

The single-cell RNA-seq (scRNA-seq) data of 3 GC tumor tissues were downloaded from GEO under accession number GSE163558 [32]. The data were analyzed in R v.4.2.1 using Seurat v.4.2.0 package for data preprocessing, UMAP nonlinear dimensionality reduction, and cell cluster recognition and clustering. According to the Cellmarker database (http://xteam.xbio.top/CellMarker/), the cell population expressing *CD8A* and *GZMK* (*granzyme K*) (cluster 1) was annotated as CD8^+^ T cells, and the cell population expressing *CD24*, *KRT19* (*keratin 19*), *KRT18* (*keratin 18*) and *EPCAM* (*epithelial cell adhesion molecule*) (cluster 2, 3, 7, 11 and 15) was annotated as tumor cells, from which tumor cell clusters with high expression (cluster 3, 7, 11 and 15) and low expression (cluster 2) of *ALKBH5* were extracted. The remaining cell clusters not covered by these markers were labeled as clusters of other cells. CellChat v.1.5.0 package was used to analyze the cell–cell contact interactions between CD8^+^ T cells and *ALKBH5* high-expression or low-expression tumor cells.

The bulk RNA-seq data of stomach adenocarcinoma with anti-PD1 immunotherapy information were collected from Sequence Read Archive (SRA) data (https://ncbi.nlm.nih.gov/sra) [33]. We used TIGER (Tumor Immunotherapy Gene Expression Resource) (http://tiger.canceromics.org/) to analyze the gene signatures for discerning therapy responses [34].

### Multiplex fluorescent immunohistochemistry (mfIHC) assay

Multiplex fluorescent immunohistochemistry assay was performed to spatially visualize and quantify the following markers: HSPA4, ALKBH5, CD58 and CD8. The Formalin-fixed paraffin-embedded GC tumor tissues were analyzed by using the Polaris® system (PerkinElmer, Waltham, MA, USA) with the customized Opal 5-color Manual IHC Kit (Panovue, Beijing, China) according to the manufacturer’s instructions. Firstly, slides were deparaffinized with xylene, rehydrated with graded ethanol. Antigen retrieval was performed in EDTA buffer (pH 8.0) for 10 min before each antibody incubation. The following antibody dilutions paired with Opal tyramide signal amplification reagent were applied in the following order for each panel: HSPA4 (1:500) with Opal 520, ALKBH5 (1: 6000) with Opal 690, CD58 (1:400) with Opal 570; CD8 (Operating fluid) with Opal 480. Slides were incubated with first primary antibody at 4 °C overnight in a moist chamber, and then incubated with paired Opal tyramide signal amplification reagent at room temperature for 1 h. After thorough washing, the slides were incubated with HRP-labeled secondary antibody at room temperature for 10 min. Repeat the above steps until the last set of antibody was stained. Finally, slides were nuclear counterstained with DAPI (Abcam, Cambridge, UK). Slides were scanned at high resolution (×20, 0.5 µM pixel) using Polaris system. Positive stain quantification and spatial cell analysis was performed using HALO 3.3 image analysis software (Indica Labs, Albuquerque, NM).

### Statistical analysis

GraphPad Prism 7.0 and R v.4.2.1 were used for data analyses. Image J was used to quantify the grayscale values of western blotting images. Normalization was performed by: values of target protein bands/values of internal control bands. For mfIHC results, we calculated the best cut-off value for the average fluorescence intensity of HSPA4 using the R package maxstat (Maximally selected rank statistics with several p-value approximations version: 0.7–25), set the minimum sample size of the group to be greater than 25%, the maximum sample number of the group to be less than 75%, and the best cut-off value is 36.8. Based on this, the patients were divided into HSPA4 upregulation and normal expression groups. Kaplan-Meier analysis with the log-rank test was performed to analyze the probability of differences in the overall survival (OS). *P* < 0.05 was considered statistically significant. One-way analysis of variance tests was performed to compare the significant differences among more than two groups. Other significant differences were analyzed by unpaired t-test. All results are presented as the mean ± SD.

## Results

### HSPA4 expression is upregulated in GC tumor tissues

**Fig. 1:**
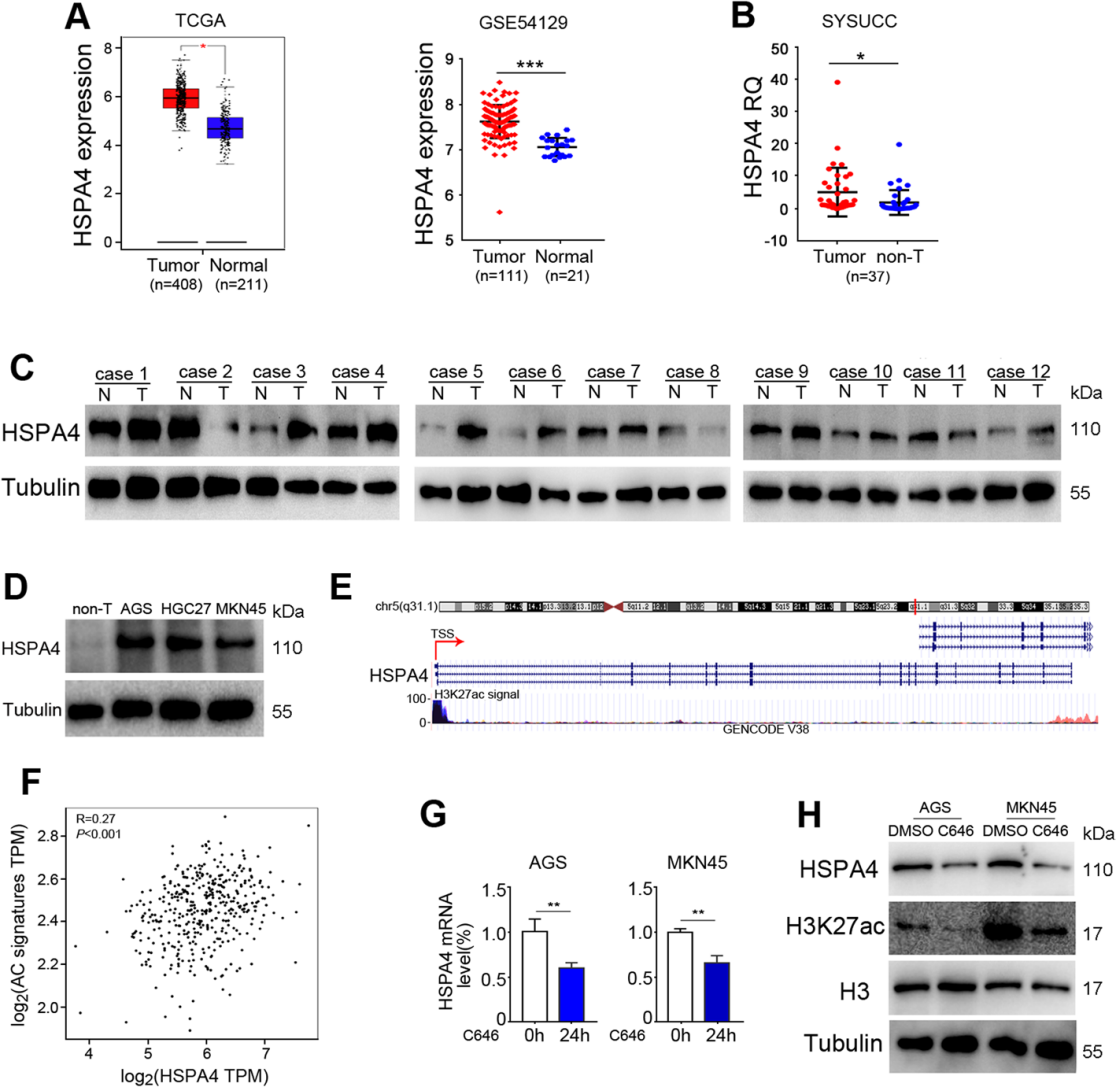
HSPA4 is upregulated in GC tissues via promoter acetylation. **A** HSPA4 was upregulated in GC tissues compared with normal tissues in TCGA and GTEx datasets (left) and GEO dataset (GSE54129) (right). **B** HSPA4 was upregulated in GC tissues compared with paired non-tumor tissues collected at SYSUCC. **C** HSPA4 protein level was determined in GC tumor tissues and paired non-tumor tissues. β-Tubulin was used as a loading control. **D** The protein level of HSPA4 was analyzed in GC cell lines and pooled non-tumor tissues (10 cases of non-tumor tissues). β-Tubulin was used as a loading control. **E** High enrichment of H3K27ac signals in the promoter region of HSPA4. **F** GEPIA analysis showed that HSPA4 expression was positively correlated with histone acetylation signature. **G**, **H** The RNA (**G**) and protein (**H**) levels of HSPA4 were detected in GC cells treated with acetyltransferase inhibitor C646 or vehicle control. β-Tubulin was used as a loading control. (*, *P* < 0.05; **, *P* < 0.01; ***, *P* < 0.001)

The analyses on The Cancer Genome Atlas (TCGA), Genotype-Tissue Expression (GTEx) and GSE54129 cohorts showed that HSPA4 was significantly upregulated in GC tissues compared with normal gastric tissues (Fig. 1A). Consistent with these findings, qRT-PCR analysis was performed on 37 pairs of GC tumor tissues and matched non-tumor tissues collected at SYSUCC. The results revealed that HSPA4 expression was higher in tumor tissues than matched non-tumor tissues (*P* < 0.05; Fig. 1B). Western blotting results indicated that HSPA4 protein level was significantly increased in 7/12 GC tissues compared with non-tumor tissues (Fig. 1C). Cell lines were also detected and HSPA4 was significantly increased in GC cells compared with pooled non-tumor tissues (Fig. 1D).

To investigate the mechanism of HSPA4 upregulation in GC, we analyzed the promoter region of HSPA4 by the UCSC genome bioinformatics website (http://genome.ucsc.edu/) and found that H3K27 acetylation (H3K27ac) signals were enriched (Fig. 1E). Consistent with that, GEPIA (Gene Expression Profiling Interactive Analysis) (http://gepia.cancer-pku.cn) results also indicated that histone acetylation signature (EP300 and CREBBP, that form a histone acetyltransferase complex) was positively correlated with HSPA4 expression in GCs (*R* = 0.27, *P* < 0.001; Fig. 1F). Two GC cell lines (AGS and MKN45) were treated with histone acetyltransferase inhibitor C646 and HSPA4 was examined. The results revealed that HSPA4 was significantly decreased in RNA and protein levels in C646-treated cells compared with DMSO-treated cells (Fig. 1G-H), suggesting that HSPA4 expression was affected by histone acetylation at H3K27. Taken together, these results indicate that HSPA4 expression is upregulated in GC tissues and histone acetylation is a reason for HSPA4 upregulation in GC tissues.

### HSPA4 upregulation promotes GC cell growth

**Fig. 2:**
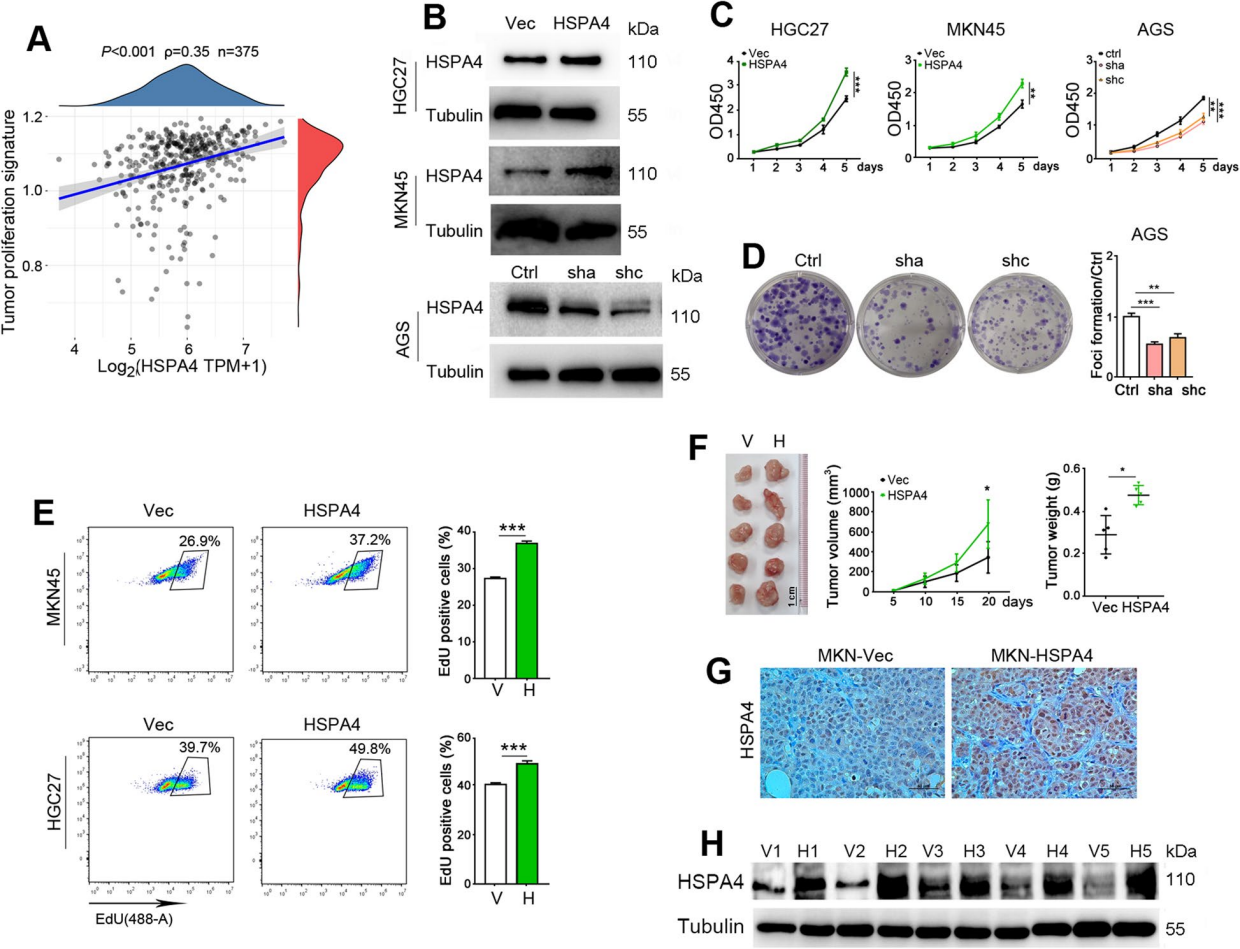
HSPA4 overexpression promotes GC cell growth. **A** HSPA4 expression was positively correlated with tumor proliferation signature in TCGA GC dataset. **B** The protein level of HSPA4 was determined in GC cells with HSPA4 overexpression or knockdown. β-Tubulin was used as a loading control. **C** The cell proliferation was determined in GC cells with HSPA4 overexpression or knockdown. **D** Foci formation results of HSPA4 knockdown AGS cells compared with control cells. **E** Flow cytometry analysis of EdU(+) cells in HSPA4-overexpressing GC cells. **F** The image, tumor growth curves and weight of xenografts formed in nude mice by injecting MKN45-HSPA4 and vector control cells subcutaneously (*n*=5). **G** Representative pictures of HSPA4 staining in xenografts formed by MKN45-HSPA4 and control cells (scale bar=50 µm). **H** HSPA4 was determined in tumors formed in 2F (V, vector; H, HSPA4). (*,*P*<0.05; **, *P*<0.01; ***, *P*<0.001)

Because the expression of HSPA4 was significantly correlated with the tumor proliferation signature in TCGA GC dataset (Fig. 2A), we established stable HSPA4-overexpressing GC cells (MKN45-HSPA4 and HGC27-HSPA4) and HSPA4 knockdown cells (AGS-sh) to explore the function of HSPA4 in GC cells (Fig. 2B). Cell proliferation assay, foci formation assay and EdU incorporation assay showed that HSPA4 overexpression promoted GC cell growth and knockdown of HSPA4 decreased cell growth (Fig. 2C-E). In vivo assay was also performed by inoculating MKN45 derivative cells (MKN-HSPA4 and MKN-Vec) subcutaneously into nude mice. The results exhibited that HSPA4 overexpression facilitated tumor growth compared with the control cells, as reflected by tumor size and weight (*P* < 0.05; Fig. 2F). HSPA4 expression was confirmed in xenografts derived from MKN-HSPA4 and MKN-Vec cells (Fig. 2G-H). These data suggest that HSPA4 promotes GC cell growth in vitro and in vivo.

### HSPA4 regulates m^6^A modification of RNA via inhibiting degradation of ALKBH5

**Fig. 3:**
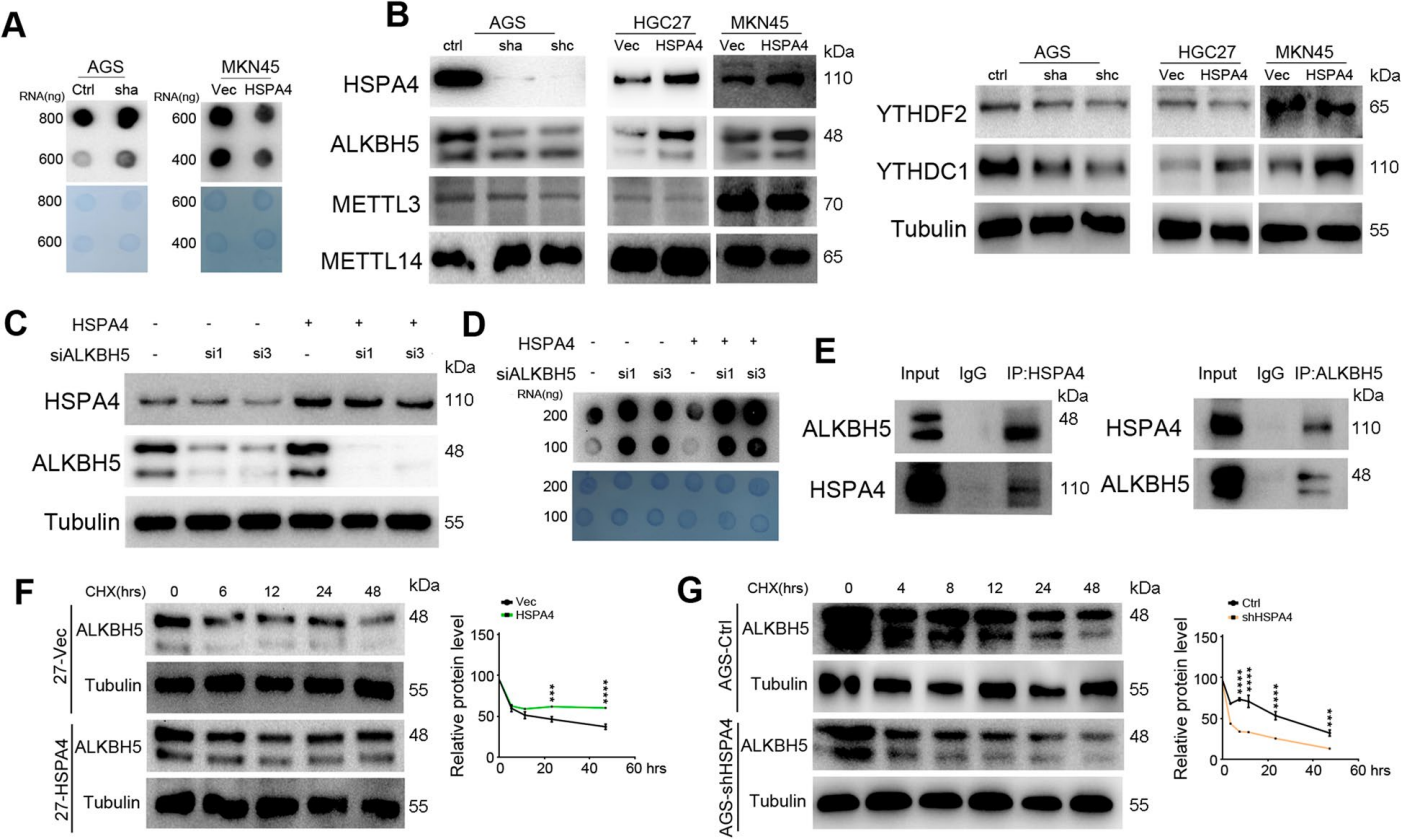
HSPA4 inhibits RNA m^6^A modification via stabilizing ALKBH5. **A** Dot blot analysis results of the total RNA of HSPA4-overexpressing or knockdown cells blotted with an anti-m^6^A antibody (*upper*). Methylene blue staining served as a loading control (*bottom*). **B** The protein levels of m^6^A methylation eraser (ALKBH5), writers (METTL3 and METTL14), and readers (YTHDF2 and YTHDC1) were determined by western blotting. β-Tubulin was used as a loading control. **C** The protein levels of HSPA4 and ALKBH5 in MKN45-HSPA4 and control cells transfected with siRNAs targeting ALKBH5. β-Tubulin was used as a loading control. **D** Total RNA was extracted from cells of **C** and m^6^A modification was examined by dot blotting (*upper*). Methylene blue staining served as a loading control (*bottom*). **E** Co-IP experiments were performed using either a HSPA4 antibody to pull down ALKBH5 or a ALKBH5 antibody to pull down HSPA4 in HGC27-HSPA4 cells, then proteins were examined. **F**, **G** The protein level of ALKBH5 was detected in HGC27-HSPA4 vs. control cells (**F**) and HSPA4 knockdown AGS (AGS-shHSPA4) vs. control cells (**G**) after cells were treated with CHX for different time. Relative protein levels of ALKBH5 were summarized in the line chart (*right*). (***, *P*<0.001; ****, *P*<0.0001)

Since m^6^A RNA methylation regulates heat shock proteins (HSPs) gene expression [35], we wondered whether HSPA4 modifies m^6^A RNA methylation. Dot blot assay was performed to examine the m^6^A RNA methylation level in HSPA4 manipulated cells. As shown in Fig. 3A, HSPA4 knockdown increased the m^6^A modification level of total RNA in GC cells, while HSPA4 overexpression decreased the m^6^A methylation level. Furthermore, some proteins related to m^6^A methylation, including writers (METTL3 and METTL14), eraser (ALKBH5) and readers (YTHDF2 and YTHDC1), were detected by western blotting. ALKBH5 and YTHDC1 were decreased in HSPA4 knockdown cells compared with control cells; whereas, the two proteins were increased in HSPA4-overexpressing cells (Fig. 3B). The RNA level of m^6^A demethylase ALKBH5 was also detected and it was not affected by HSPA4 overexpression or knockdown (Supplementary Fig. 1). To further explore the role of ALKBH5 in RNA methylation regulated by HSPA4, siRNAs targeting ALKBH5 were transfected into HSPA4-overexpressing MKN45 cells (Fig. 3C). Dot blot results revealed that silencing ALKBH5 could reverse the decreased level of m^6^A RNA methylation in MKN45-HSPA4 cells (Fig. 3D). Because the protein level of ALKBH5 was changed and RNA level remained unchanged in HSPA4-overexpressing or knockdown GC cells, we wondered whether HSPA4 could stabilize the protein level of ALKBH5. Co-IP experiments indicated that HSPA4 could interact with ALKBH5 in HGC27-HSPA4 cells (Fig. 3E). We used cycloheximide to inhibit protein synthesis and examined ALKBH5 at different time points by western blotting. The protein degradation of ALKBH5 was decreased in HSPA4-overexpressing HGC27 cells (Fig. 3F). In consistent with this, ALKBH5 degraded more rapidly in HSPA4 knockdown AGS cells than in control cells (Fig. 3G). Taken together, HSPA4 regulates the m^6^A modification level of RNA by reducing ALKBH5 degradation.

### HSPA4 negatively regulates CD58 expression via ALKBH5

**Fig. 4:**
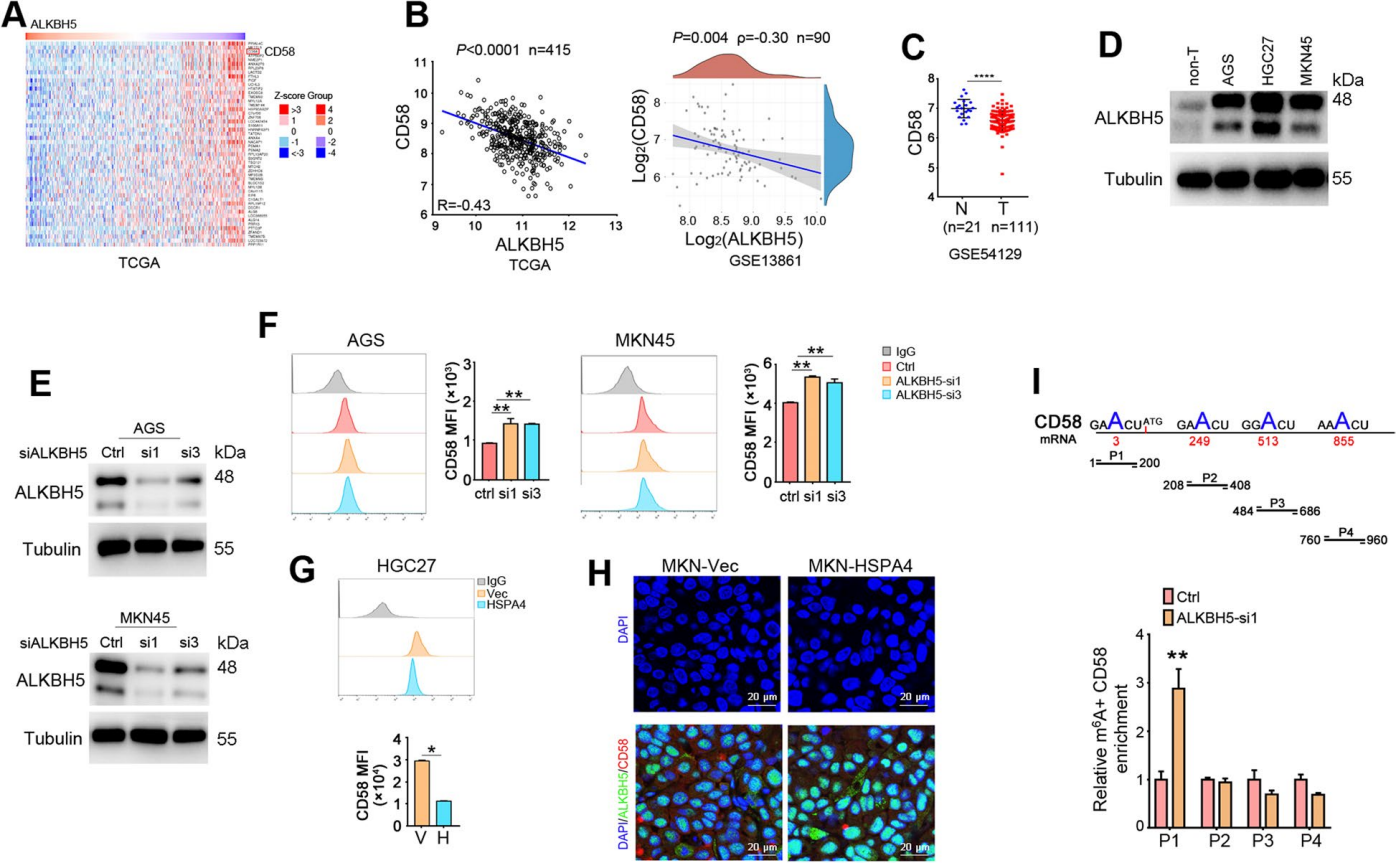
ALKBH5 negatively regulates CD58 via m^6^A RNA demethylation. **A** Heat map showed the expression of ALKBH5 was negatively correlated with CD58 in TCGA GC database. **B** Scatter plot showed the correlation between the expression of ALKBH5 and CD58 in patients with GC in TCGA (*left*) and GSE13861 (GEO, *right*). **C** CD58 was evaluated in GC tumor tissues and normal gastric tissues from GSE54129 cohort. **D** The protein level of ALKBH5 was analyzed in GC cell lines and pooled non-tumor tissues (10 cases of non-tumor tissues). β-Tubulin was used as a loading control. **E** The protein level of ALKBH5 was determined in ALKBH5 knockdown AGS and MKN45 cells. β-Tubulin was used as a loading control. **F**, **G** The expression of CD58 in ALKBH5 knockdown cells (AGS and MKN45) (**F**) and HGC27-HSPA4 cells (**G**) was analyzed by flow cytometry. Representative pictures and mean fluorescence intensity (MFI) of CD58 was summarized. **H** Immunofluorescence images of ALKBH5 (green) and CD58 (red) in xenografts formed by MKN45-HSPA4 and control cells in nude mice (Figure 2F) (scale bar=20 µm). **I** The primers were designed according to the possible m^6^A methylation sites on CD58 mRNA (*upper*). MeRIP-qPCR was performed in ALKBH5-KD AGS cells and control cells and m^6^A+ CD58 fragments enrichments were assayed. (*, *P*<0.05; **, *P*<0.01; ****, *P*<0.0001)

ALKBH5 is an essential m^6^A demethylase that controls many biological processes by modulating transcription, translation and cellular localization of RNA [36]. By using correlation analysis in TCGA GC data, we screened out the top 50 genes that were correlated negatively with ALKBH5 and CD58 was identified (Fig. 4A). Negative correlation between CD58 and ALKBH5 was verified in TCGA and GEO datasets (GSE13861) (Fig. 4B). CD58 is a ligand of CD2 that expresses in T lymphocytes and enhances T cells activation [37]. We also observed that CD58 was downregulated in GC tumor tissues compared with normal gastric tissues in GEO cohort (GSE54129) (Fig. 4C). To evaluate whether ALKBH5 could regulate CD58, we first examined the protein level of ALKBH5 in GC cell lines and found that it was upregulated in GC cell lines compared with pooled non-tumor gastric tissues (Fig. 4D). GC cell lines, AGS and MKN45, were used to knockdown ALKBH5 with siRNAs (Fig. 4E) and CD58 was assayed. Flow cytometry showed that CD58 expression was increased in ALKBH5 knockdown cells compared with control cells (Fig. 4F). CD58 was also examined in HGC27-HSPA4 cells and it was decreased in the HSPA4 upregulated cells (Fig. 4G). To further confirm the interactions among HSPA4, ALKBH5 and CD58, the xenografts formed by MKN45-HSPA4 and control cells in nude mice (Fig. 2F) were stained with ALKBH5 and CD58 antibodies. The immunofluorescence results demonstrated increased ALKBH5 and decreased CD58 in MKN45-HSPA4 xenografts compared with the xenografts formed by control cells (Fig. 4H).

We next investigated mechanism of ALKBH5 involvement in CD58 regulation. QRT-PCR results revealed that the RNA level of CD58 was increased in ALKBH5 knockdown cells (Supplementary Fig. 2). We asked whether ALKBH5 could regulate CD58 via m^6^A RNA methylation. By searching SRAMP, a sequence-based m^6^A modification site predictor website (http://www.cuilab.cn/sramp), we found that CD58 mRNA might be modified at positions of 3, 249, 513 and 855 nt of mRNA sequence (Fig. 4I). Primers were designed based on the prediction sites and Me-RIP assays were performed on ALKBH5-KD and control cells. The results displayed that P1 fragment (encircling the position 3 nt of CD58 mRNA) was significantly enriched by m^6^A-specific antibody in ALKBH5 knockdown cells compared with control cells (Fig. 4I). Accordingly, these results suggest that HSPA4 negatively regulates CD58 via demethylation effect of ALKBH5 on CD58 mRNA sequence.

### HSPA4/ALKBH5/CD58 axis inhibits the cytotoxicity of CD8 ^+^ T cells

**Fig. 5:**
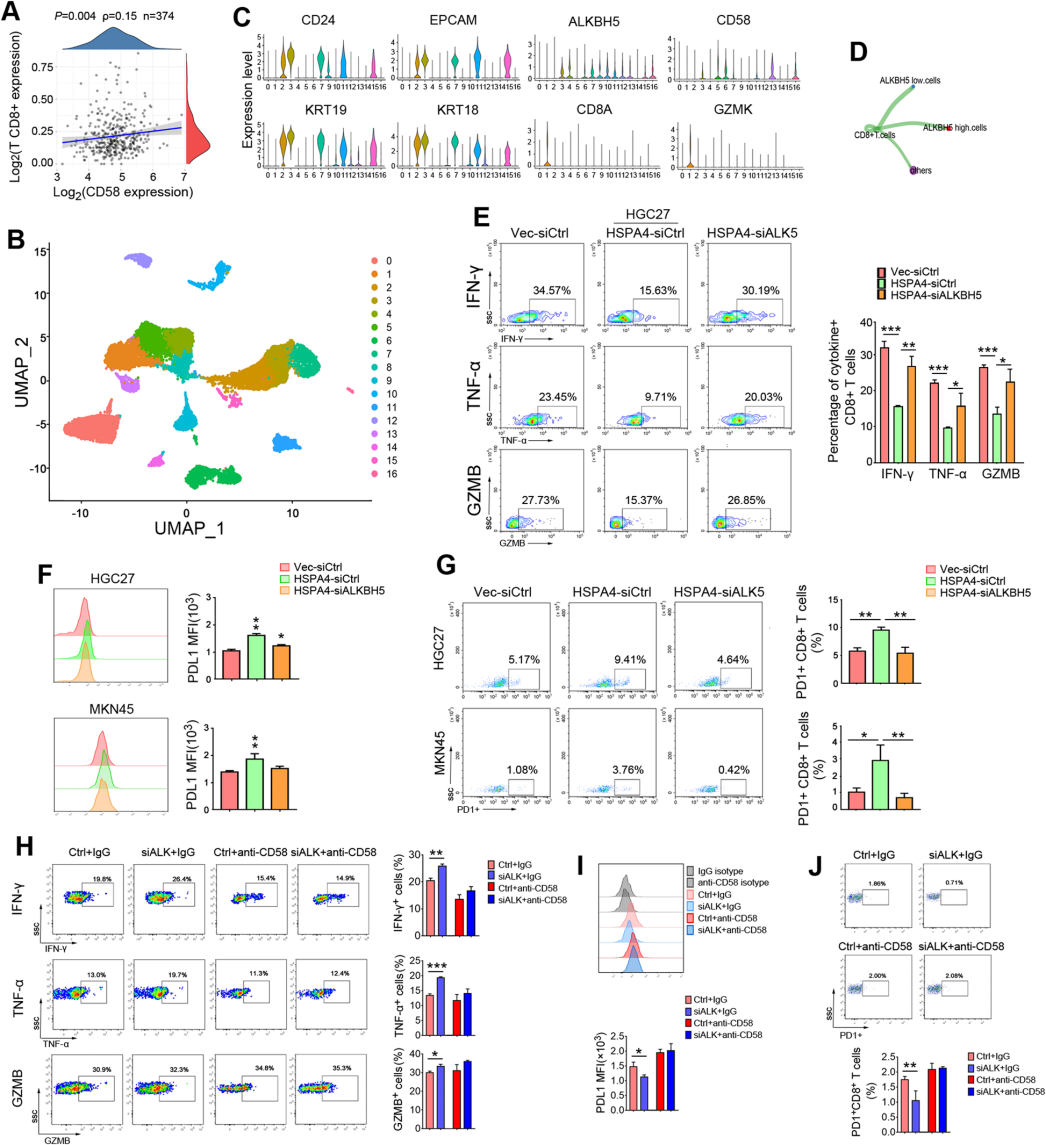
HSPA4/ALKBH5 inhibits cytotoxicity of CD8^+^ T cells by downregulating CD58 in GC cells. **A** The correlation between CD58 expression and immune score of CD8^+^ T cells was analyzed within GC patients (spearman’s correlation). **B** UMAP plot of cells from 3 cases of GC tumor tissues showing the formation of 17 main clusters from 15729 cells (GSE163558). **C** Violin plots showing the expression distribution of marker genes in tumor epithelial cell clusters (*CD24*,*KRT19*, *KRT18* and *EPCAM*), ALKBH5 expression cell clusters (*ALKBH5*) and CD8^+^ T cell clusters (*CD8A* and *GZMK*). **D** Cell–cell contact interactions analysis showing strength of interactions between ALKBH5 high-expression or low-expression tumor cells and CD8^+^ T cells. **E** HGC27 cells with HSPA4 overexpression and ALKBH5 knockdown were co-cultured with CD8^+^ T cells for 16 h. Intracellular staining of IFN-ɤ, TNF-α and GZMB were determined by flow cytometry. Representative images and summary of IFN-ɤ, TNF-α and GZMB positive T cells were listed.**F**,**G** The GC cells with HSPA4 overexpression and ALKBH5 knockdown were co-cultured with CD8^+^ T cells for 72 **h**. The expression levels of PDL1 on the surface of tumor cells (**F**) and PD1 on the surface of CD8^+^ T cells (**G**) were analyzed by flow cytometry. Representative images and summaries of PDL1 MFI and PD1^+^ T cells ratio were listed.**H** ALKBH5-KD AGS and control cells were incubated with anti-CD58 or isotype control IgG for 12 h, followed by co-culture with CD8^+^ T cells for 16 h. Intracellular staining of IFN-ɤ, TNF-α and GZMB were determined by flow cytometry. Representative images and ratios of IFN-ɤ, TNF-α and GZMB positive CD8^+^ T cells were summarized. **I**,**J** ALKBH5-KD AGS and control cells were incubated with anti-CD58 or IgG antibodies for 12 h, followed by co-culture with CD8^+^ T cells for 72 h. Expression of PDL1 on tumor cells (**I**) and PD1 on CD8^+^ T cells (**J**) were analyzed by flow cytometry. Representative images and results of PDL1 MFI (**I**) and PD1^+^CD8^+^ T cells ratio (**J**) were summarized. (*, *P*<0.05; **, *P*<0.01; ***, *P*<0.001)

 We next analyzed TCGA GC dataset and found that the expression of CD58 was positively correlated with the immune score of CD8^+^ T cells in GC tissues (*P* = 0.004) (Fig. 5A). A single-cell sequencing dataset (GSE163558) including 3 GC tissues were obtained from GEO and 15,729 cells in total were pooled together for subsequent analysis. Seventeen cell clusters were visualized using uniform manifold approximation and projections (UMAP) analysis (Fig. 5B). We defined clusters 2, 3, 7, 11 and 15 as epithelial tumor cells through marker genes including *CD24*, *KRT19*, *KRT18* and *EPCAM*. The epithelial tumor cells were partitioned into two clusters, including ALKBH5 high-expression clusters (3, 7, 15,11 and ) and low-expression cluster [2] (Fig. 5C). Cluster 1 was assigned to CD8^+^ T cells marked with *CD8A* and *GZMK* (Fig. 5C). Cell–cell contact interactions between ALKBH5 high-expression or low-expression tumor cells with CD8^+^ T cells were analyzed by CellChat v.1.5.0 package. The results indicated that cell–cell contact interactions between ALKBH5 low-expression cells and CD8^+^ T cells was stronger than that between ALKBH5 high-expression cells and CD8^+^ T cells (Fig. 5D).

To assess whether HSPA4/ALKBH5/CD58 axis affects CD8^+^ T cells cytotoxicity, we overexpressed HSPA4 in GC cells (HGC27-HSPA4 and MKN45-HSPA4), then knocked down ALKBH5 with siRNA targeting ALKBH5. Coculture cell models were established by coculturing GC cells with CD8^+^ T cells isolated from PBMC of healthy donors. CD8^+^ T cells were then stained with antibodies and examined by flow cytometry. The production of IFN-γ, TNF-α and Granzyme B (GZMB) was drastically decreased in CD8^+^ T cells cocultured with HGC27-HSPA4 cells (Fig. 5E). When HGC27-HSPA4 cells were transfected with siRNA against ALKBH5 and cocultured with CD8^+^ T cells, the effect was reversed in CD8^+^ T cells (Fig. 5E). The experiments were repeated in CD8^+^ T cells cocultured with MKN45 derivative cells and similar results were observed (Supplementary Fig. 3). Furthermore, we detected PDL1 expression in tumor cells and PD1 in CD8^+^ T cells collected from the coculture experiments. The flow cytometry analyses demonstrated that PDL1 was significantly upregulated in HSPA4-overexpressing GC cells and decreased after ALKBH5 was silenced (Fig. 5F). In addition, the percentage of PD1-positive CD8^+^ T cells was increased in CD8^+^ T cells cocultured with HSPA4-overexpressing GC cells and decreased after ALKBH5 was silenced (Fig. 5G).

To further validate the role of CD58 in the signaling axis, we blocked CD58 with antibody in ALKBH5 knockdown AGS cells. The cells were cocultured with CD8^+^ T cells and flow cytometry analysis was performed. The percentages of IFN-γ, TNF-α or GZMB positive CD8^+^ T cells were significantly increased in CD8^+^ T cells cocultured with ALKBH5-KD GC cells (Fig. 5H). However, when CD58 was blocked, the increases of IFN-γ, TNF-α or GZMB positive CD8^+^ T cells were attenuated (Fig. 5H). In addition, PDL1 on GC cells and PD1 on cocultured CD8^+^ T cells were also examined. Mean fluorescence intensity of PDL1 was decreased in ALKBH5-KD GC cells and the percentage of PD1^+^CD8^+^ T cells was decreased in cocultured T cells, whereas the decreases were dampened when CD58 was blocked (Fig. 5I-J).

### Clinical significance of HSPA4/ALKBH5/CD58 axis in GC tumor tissues

**Fig. 6:**
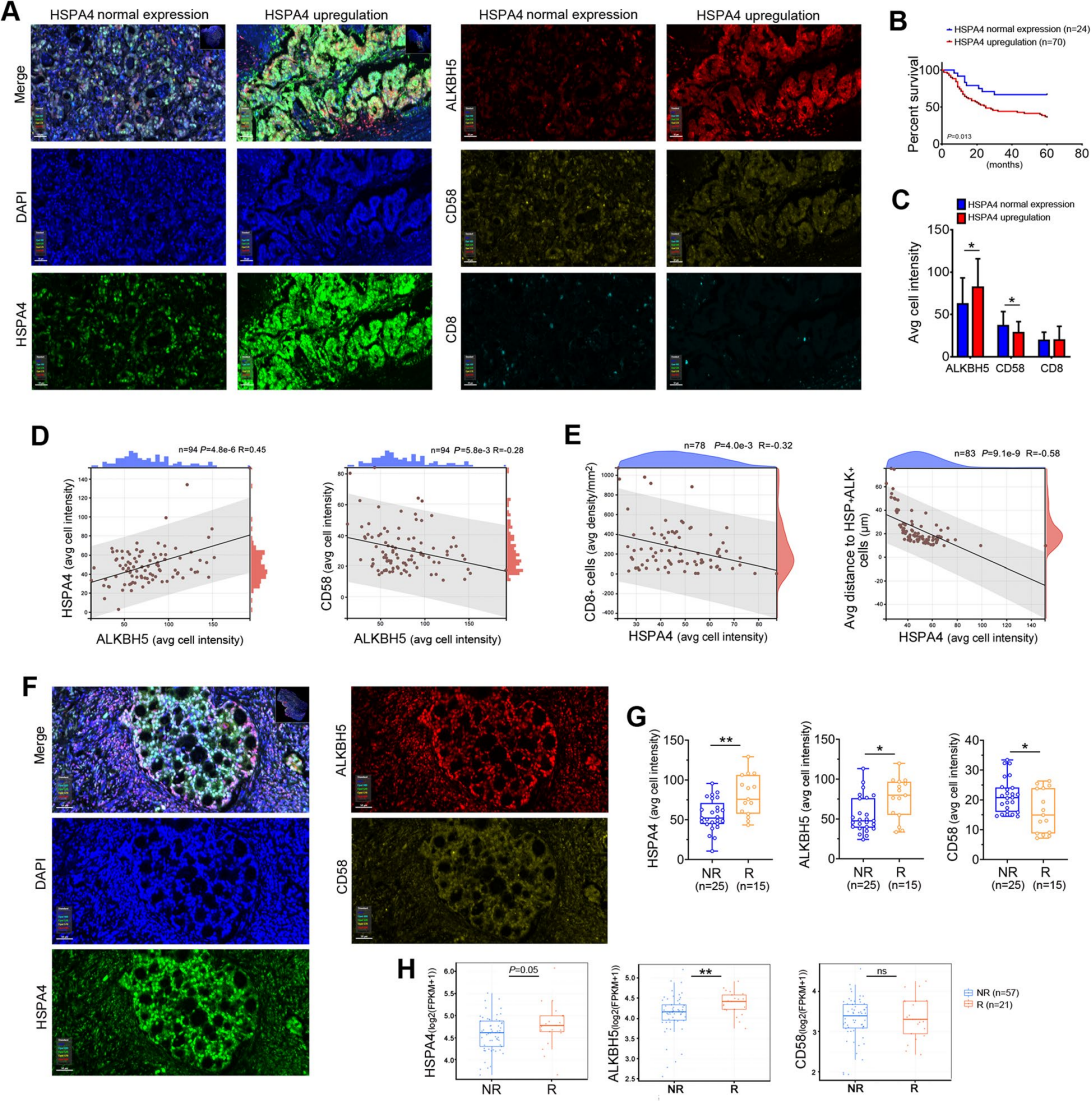
HSPA4/ALKBH5/CD58 axis in patients with GC. **A** Representative pictures of mfIHC for HSPA4, ALKBH5, CD58 and CD8 in GC patients’ specimens. Cell nuclei were counterstained with DAPI (scale bar=50 µm). **B** The survival curve showed that HSPA4 upregulation correlated with poor five-year survival with GC patients. **C** The average fluorescence intensity of ALKBH5, CD58 and CD8 in tumor tissues with different HSPA4 expression levels were summarized. **D** The correlation between average fluorescence intensity of ALKBH5 and HSPA4 (*left*), as well as ALKBH5 and CD58 (*right*) in tumor tissues were analyzed by Pearson’s correlation. **E** The correlation between average fluorescence intensity of HSPA4 and average density of CD8^+^ T cell (*left*), as well as fluorescence intensity of HSPA4 and distance from CD8^+^ T cells to ALKBH5^+^HSPA4^+^ cells (*right*) were analyzed by Pearson’s correlation. **F** Representative pictures of mfIHC for HSPA4, ALKBH5 and CD58 in GC patients receiving PD1 blockade therapy (SYSUCC). Cell nuclei were counterstained with DAPI (scale bar=50 µm). **G** Box plots with the expression of HSPA4, ALKBH5 and CD58 from responders and non-responders of PD1 blockade therapy (SYSUCC) (Wilcoxon rank-sum test). **H** Box plots showed the RNA levels of HSPA4, ALKBH5 and CD58 from responders and non-responders of PD1 blockade therapy (SRA data (https://ncbi.nlm.nih.gov/sra) analyzed by TIGER) (Wilcoxon rank-sum test). (*, *P*<0.05; **, *P*<0.01; ***, *P*<0.001)

To further explore the clinical significance of HSPA4/ALKBH5/CD58 axis in GC tissues, multiplex fluorescent immunohistochemistry assay was carried out and HSPA4, ALKBH5, CD58 and CD8 were examined (Fig. 6A). GC tissue samples were divided into HSPA4 upregulation group (*n* = 70) and normal expression group (*n* = 24) according to the cut-off fluorescence intensity of HSPA4. Kaplan-Meier survival analysis revealed that HSPA4 upregulation was significantly associated with poor 5-year survival in patients with GC (*P* = 0.013, Fig. 6B). Multivariate analysis also demonstrated that HSPA4 upregulation was an independent prognosis factor (Table 1). The average cell fluorescence intensity of ALKBH5, CD58 and CD8 were evaluated and the results exhibited that ALKBH5 was higher in HSPA4 upregulation group compared with HSPA4 normal expression group, whereas CD58 was lower in HSPA4 upregulation group (*P* < 0.05, Fig. 6C). Correlation analysis results demonstrated that ALKBH5 correlated positively with HSPA4 while negatively with CD58 in GC tumor tissues (Fig. 6D). To quantify the infiltration of CD8^+^ T cells in tumor mass, spatial distribution of CD8^+^ T cells was measured within 100 μm around tumor cells. Although we did not observe the difference of CD8 intensity between HSPA4 upregulation group and normal expression group (Fig. 6C), the spatial distribution results indicated that HSPA4 was negatively correlated with the average distribution density of CD8^+^ T cells and the average distance from CD8^+^ T cells to HSPA4^+^ALKBH5^+^ tumor cells (Fig. 6E). Taken together, these results suggest that the upregulation of HSPA4/ALKBH5/CD58 signaling axis in GC tumor cells negatively regulates the infiltration of CD8^+^ T cells in GC tumor mass.

**Table 1:** Univariate and multivariate analyses of prognostic variables in patients with GC

Clinicopathologic characters	Univariate analysis^a^		Multivariate analysis^a^	
	HR(95% CI)	* P* value	HR(95% CI)	* P* value
Sex (female vs. male)	1.590 (0.610–4.139)	0.343		
Age (> 60 vs. ≤60)	1.175 (0.476–2.901)	0.726		
Differentiation (low + middle vs. high)	1.679 (0.250-11.289)	0.594		
TNM (III-IV vs. I-II)	2.254 (0.904–5.619)	0.081		
HSPA4 upregulation	5.119 (1.688–15.529)	0.008	2.483(1.170–5.247)	0.018

*HR* hazard ratio

^a^Cox regression analysis

### The clinical significance of HSPA4 upregulation in anti-PD1 checkpoint therapy

Considering that HSPA4 upregulation in GC cells decreased the percentage of PD1^+^CD8^+^ T cells in cocultured CD8^+^ T cells, we asked whether HSPA4 upregulation could affect the therapy effect of anti-PD1 checkpoint therapy. Tumor tissues were collected from 40 GC patients receiving anti-PD1 immunotherapy combined with chemotherapy. The patients were divided into responder (R group, *n* = 15) and non-responder (NR group, *n* = 25) groups according to clinical outcome. We compared the protein levels of HSPA4, ALKBH5 and CD58 in tumor tissues between responders and non-responders by using mfIHC (Fig. 6F). As shown in Fig. 6G, increased HSPA4 (*P* < 0.01), ALKBH5 (*P* < 0.05) and decreased CD58 (*P* < 0.05) were observed in tumor tissues of responders compared with non-responders. The bulk RNA-seq data of stomach adenocarcinoma with PD1 immunotherapy information from SRA were analyzed by TIGER, an online software for tumor immunotherapy gene expression resource. The results demonstrated increased RNA levels of HSPA4 (*P* = 0.05) and ALKBH5 (*P* < 0.01) in responders compared with non-responders (Fig. 6H). For CD58 RNA level, no significant difference was observed between responders and non-responders (Fig. 6H). Taken together, these results indicate that HSPA4 upregulation is a promising biomarker for predicting the effect of PD1 blockade therapy in patients with GC.

**Fig. 7:**
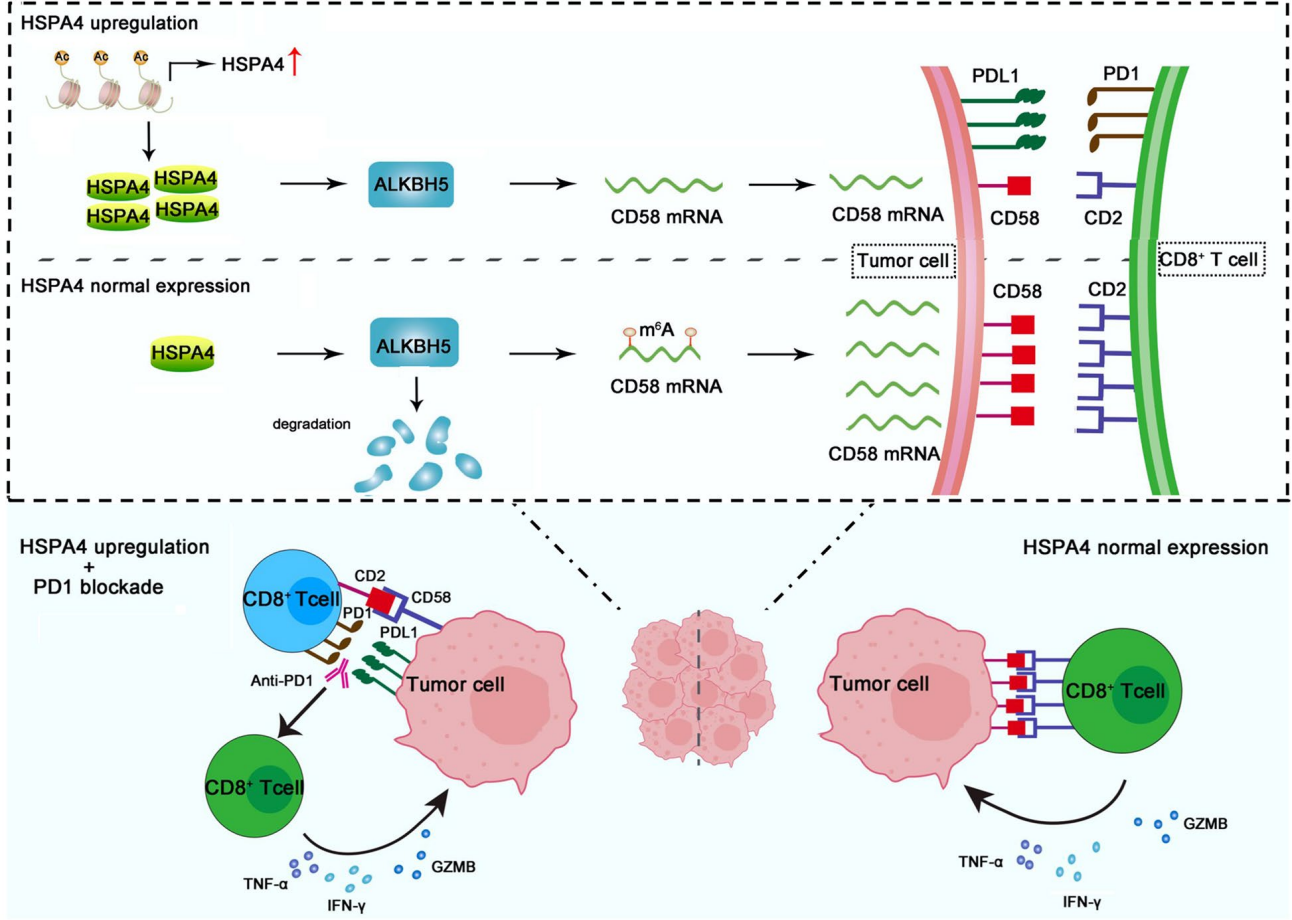
Proposed model depicting HSPA4 modulating immune evasion and response to PD1 blockade. HSPA4 is upregulated via promoter acetylation in GC tumor tissues. HSPA4 upregulation inhibits ALKBH5 degradation, leading to downregulation of CD58 in GC cells via m^6^A demethylation activity of ALKBH5, PDL1 upregulation in tumor cells and impairing cytotoxicity of CD8^+^ T cells, finally to immune evasion of tumor cells. Cytotoxicity of CD8^+^ T cells is re-activated in the presence of anti-PD1 checkpoint therapy

Together these data suggest that histone acetylation upregulates HSPA4 expression in GC cells. HSPA4 overexpression upregulates ALKBH5 via increasing its protein stability, leading to decrease of CD58 of tumor cells and increase of PD1/PDL1 axis, finally to impair the toxicity of CD8^+^ T cells and immune evasion of GC cells. When tumor cells with high expression of HSPA4 are treated with PD1 blockade therapy, CD8^+^ T cells are re-activated and cell toxicity is restored, then tumor cells respond better to PD1 checkpoint blockade therapy (Fig. 7).

## Discussion

Heat shock proteins (HSPs) represent a prototypical family of molecular chaperone genes, playing a crucial role in tumor progression and being regarded as targets for anticancer therapies [38]. Accumulating evidences have proved that HSPs prompt the immune system to react to prevailing adverse cellular conditions and HSPs are implicated in both pro-inflammatory and anti-inflammatory responses [39]. Their effects on immunomodulatory function depend on a number of aspects such as concentration of the respective HSP species [39]. Our study suggests that HSPA4 overexpression in GC cells exerts immunomodulatory functions and impairs the toxicity of CD8^+^ T cells, finally leading to immune evasion of GC cells.

HSPA4 is located at 5q31.1, which is not a common chromosomal amplification region in gastric cancer [40] Then we analyzed the promoter methylation levels in GC tissues and normal gastric mucosal tissues by searching online software SMART (http://www.bioinfo-zs.com/smartapp). No significant difference exists between GC tissues and normal mucosal tissues. Meanwhile, histone acetylation signals are enriched in HSPA4 promoter region by bioinformatics analyses and our study demonstrates that H3K27ac modification plays an important role in HSPA4 overexpression in GC tissues.

N^6^-methylation of adenosine (m^6^A) in RNA regulates many pathophysiological processes. Previous work has found that YTHDC1, a reader of N^6^-methyladenosine RNA modification, binds to m^6^A-modified HSP transcripts and promotes expression of HSPs [41]. In this study, we found that HSPA4 could regulate m^6^A modification level of RNA in GC cells and the protein levels of ALKBH5 and YTHDC1 changed significantly. ALKBH5, a m^6^A demethylase, modulates target gene expression, generates an immunosuppressive tumor microenvironment and contributes to the efficacy of immunotherapy in melanoma, colorectal cancer and glioblastoma [42, 43]. It is also a molecular target to boost immune checkpoint blockade therapy in colorectal cancer [44]. Our research demonstrates that HSPA4 overexpression increases the protein stability of ALKBH5 in GC cells.

CD58 is a co-stimulatory ligand for CD2, which is expressed mainly on CD8^+^ T cells and NK cells [37]. Loss of CD58 leads to decreased T cell engager-mediated cytotoxicity, T cell activation and antitumor efficacy [45]. CD58 mutation or downregulated expression is common in melanoma and hematological tumors, and downregulation of CD58 is associated with immune evasion in melanoma and that disruption of CD58 in tumor cells confers functional impairment of CAR T cells [46–48]. Previous study has shown that CMTM6 is critical for CD58 stability and upregulation of PDL1 upon CD58 loss in melanoma [49]. Here we show a different mechanism in gastric cancer: CD58 is negatively posttranscriptionally regulated by HSPA4/ALKBH5 via m^6^A demethylation.

By coculturing tumor cells with CD8^+^ T cells, we demonstrate that HSPA4 upregulation suppresses the cytotoxicity of CD8^+^ T cells. Additionally, upregulation of HSPA4 increases PDL1 expression in GC cells and the ratio of PD1^+^CD8^+^ T cells in cocultured CD8^+^ T cells. These effects can be retarded by silencing ALKBH5 in GC cells. It was reported that CD58 loss upregulated PDL1 expression in melanoma [48], in addition, ALKBH5 deficiency enriched m^6^A modification in the 3’UTR region of PDL1 mRNA and promoted its degradation in intrahepatic cholangiocarcinoma [50]. In our research, PDL1 is decreased when ALKBH5 is silenced in GC cells. However, when CD58 is blocked with antibody, the decrease of PDL1 is retarded, as well as the cytotoxicity of cocultured CD8^+^ T cells. These results suggest that CD58 plays an important role in HSPA4 overexpression induced immunosuppression microenvironment in GC.

Since there is no mouse homolog of CD58 in pre-clinical mouse models [51, 52], syngeneic models cannot be used to investigate tumor cells-immune cells interactions and responses to immune checkpoint inhibitors in vivo. We confirmed the HSPA4/ALKBH5/CD58 axis in clinical GC samples. The prognosis value of HSPA4 was evaluated in GC patients without pre- or post-surgery treatment. HSPA4 upregulation is associated with poor clinical outcome of patients with GC. In addition, ALKBH5 is upregulated and CD58 is downregulated in tumor tissues with HSPA4 upregulation. CD8^+^ T cells infiltration is also decreased in tumor tissues with HSPA4 upregulation. These results suggest the immunomodulatory role of HSPA4/ALKBH5/CD58 axis in GC tumor tissues.

As PD1 checkpoint blockade plus chemotherapy has been approved as the first-line treatment for gastro-esophageal cancer patients in many countries [4, 8], we also evaluated HSPA4 expression in responders and non-responders of GC patients receiving PD1 checkpoint blockade combined with chemotherapy. The results demonstrate that HSPA4 expression is higher in responders compared with non-responders, suggesting that HSPA4 upregulation might be a potential biomarker for predicting better responses to the combined therapy in patients with GC.

Collectively, our research contributes three major advancements. Firstly, we identify that HSPA4 is upregulated in GC tumor tissues and the upregulation of HSPA4 is positively associated with poor survival in patients with GC. Secondly, we unveil a novel mechanism of immune evasion of GC cells: HSPA4 overexpression in GC cells decreases CD58 via ALKBH5/CD58 pathway, activates PD1/PDL1 axis, decreases cytotoxicity and infiltration of CD8^+^ T cells and induces tumor immunosuppressive microenvironment. Thirdly, we explore the clinical relevance of HSPA4 in the sensitivity to immune checkpoint blockade therapy. HSPA4 upregulation might be a promising biomarker for predicting the sensitivity to the immune checkpoint inhibitor therapy in patients with GC. Our study helps to identify GC patients who might benefit from immune checkpoint blockade therapy.

### Supplementary Information

**Supplementary Material 1.**

## Data Availability

The dataset supporting the conclusions of this article is(are) included within the article and its additional file.

## Abbreviations

GC: Gastric cancer
HSPA4: Heat shock protein family A (Hsp70) member 4
ALKBH5: AlkB homolog 5,RNA demethylase
ATCC: America type culture collection
PBMC: Peripheral blood mononuclear cell
EdU: 5-ethynyl-2’-deoxyuridine
qRT-PCR: quantitative real time RT-PCR
IF: Immunofluorescence
DAPI: 4′,6 diamidino-2-phenylindole
co-IP: coimmunoprecipitation
Me-RIP: Methylated RNA immunoprecipitation
mfIHC: multiplex fluorescent immunohistochemistry
m^6^A: N6-methyladenosine
METTL3: Methyltransferase 3,N6-adenosine-methyltransferase complex catalytic subunit
METTL14: Methyltransferase 14,N6-adenosine-methyltransferase subunit
YTHDF2: YTH N6-methyladenosine RNA binding protein F2
YTHDC1: YTH N6-methyladenosine RNA binding protein C1
GZMK: Granzyme K
KRT19: Keratin 19
KRT18: Keratin 18
EPCAM: Epithelial cell adhesion molecule
PBS: Phosphate buffered saline
scRNA-seq: single-cell RNA-seq
SRA: Sequence Read Archive
TIGER: Tumor Immunotherapy Gene Expression Resource
OS: Overall survival
GEO: Gene Expression Omnibus
TCGA: The Cancer Genome Atlas
GTEx: Genotype-Tissue Expression
GEPIA: Gene Expression Profiling Interactive Analysis
CHX: Cycloheximide
MFI: Mean fluorescence intensity

## Declarations

All animal experiments were approved by the Animal Ethics Committee at Sun Yat-sen University Cancer Center (SYSUCC) with the num. L025501202212012.
