# Supplementary material for: HSPA4 upregulation induces immune evasion via ALKBH5/CD58 axis in gastric cancer
Source: J Exp Clin Cancer Res. 2024 Apr 8;43:106. doi: 10.1186/s13046-024-03029-4 (PMC11000359; doi:10.1186/s13046-024-03029-4)
Supplement: Supplementary file 1 — Supplementary Material 1. [file 13046_2024_3029_MOESM1_ESM.docx]

**Supplementary information**

## Primers, shRNAs and siRNAs:

| qPCR primers | |
| --- | --- |
| HSPA4 | Forward: 5’ GCCAGGTTCTTAGCACATCC 3’  Reverse: 5’ GAGAGCAAGCAGACCACATC 3’ |
| ALKBH5 | Forward: 5’ GTAGAGGTGGTGGAGCAGAG 3’  Reverse: 5’ GGCAGGTGAGAAGTGGAGAA 3’ |
| CD58 | Forward: 5’ GCACTGCTTTGGTTTCATCA 3’  Reverse: 5’ GCTTGGTACATGGAAAGTTACA 3’ |
| 18S | Forward: 5’ GTAACCCGTTGAACCCCATT 3’  Reverse: 5’ CCATCCAATCGGTAGTAGCG 3’ |
| Me-RIP primers | |
| CD58-P1 | Forward: 5’ TGTGTCAGGTAGCCTCACTATC 3’  Reverse: 5’ TGGCGATTCCATTTCATACTCA 3’ |
| CD58-P2 | Forward: 5’ ACAGTGTACTCTTAGCAATCCA 3’  Reverse: 5’ GCTTGGGATACAGGTTGTCAA 3’ |
| CD58-P3 | Forward: 5’ CAGAAGATGAAGACAACAGCAT 3’  Reverse: 5’ CTCAAATGAGAAATCAGATGGC 3’ |
| CD58-P4 | Forward: 5’ TAGTAAGTGATGGGCTGGGATT 3’  Reverse: 5’ GAGGTGGAGGGAGTGTATTGTA 3’ |
| shRNAs targeting HSPA4 | |
| shHSPA4-a  shHSPA4-c | 5’ CCAATCGAGAATCAGCTATTA 3’  5’ GGTCCTAAGAATCGTTCAATT 3’ |
| siRNAs targeting ALKBH5: | |
| siALKBH5-1  siALKBH5-3 | 5’ GATCGCCTGTCAGGAAACA 3’  5’ GCTGCAAGTTCCAGTTCAA 3’ |

## Antibodies:

| **Antibody** | **Company** | **Catalog Number** |
| --- | --- | --- |
| HSPA4 | Novus | NBP2-67817 |
| Tublin | Proteintech | 10094-1-AP |
| GAPDH | Proteintech | 10494-1-AP |
| ALKBH5 | Abcam | ab195377 |
| CD58 | Abcam | ab171087 |
| METTL3 | Affinity | DF12020 |
| METTL14 | Cell Signaling Technology | #51104S |
| YTHDC1 | Cell Signaling Technology | # 77422S |
| YTHDF2 | Affinity | DF12182 |
| N6-methyladenosine | Novus | NBP2-50525 |
| Mouse-IgG | Cell Signaling Technology | #7076 |
| Rabbit-IgG | Cell Signaling Technology | #7074 |
| anti-human CD8 | Biolegend | 344750 |
| anti-human IFN-γ | Biolegend | 502509 |
| anti-human TNF-α | Biolegend | 502923 |
| anti-human GZMB | MACS | 130-101-349 |
| anti-human CD3ε | Biolegend | 300332 |
| anti-human CD28 | Biolegend | 302934 |
| anti-human PD1 | Biolegend | 4286088 |
| anti-human PD-L1 | Biolegend | 345507 |
| anti-human CD8 | Zhong shan Goldenbridge | ZA-0508 |
| rat IgG2a isotype | Neobioscience | BE0089 |

## Reagents:

| **Reagent** | **Company** | **Catalog N.** |
| --- | --- | --- |
| C646 | Selleck | S7152 |
| cycloheximide | Santa Cruz | 3508B |
| EdU | RiBoBio | C10338-3 |
| MeRIP m^6^A Transcriptome Profiling Kit | RiBoBio | C11051-1 |
| Human IFN-gamma Recombinant Protein | PeproTech | 300-02 |
| Human IL-2 Recombinant Protein | PeproTech | 200-02 |
| Multiplex fluorescent IHC kit -5-color TSA-RM-2759 | Pavonue | 10002100050 |

**Supplementary figure legends:**

**
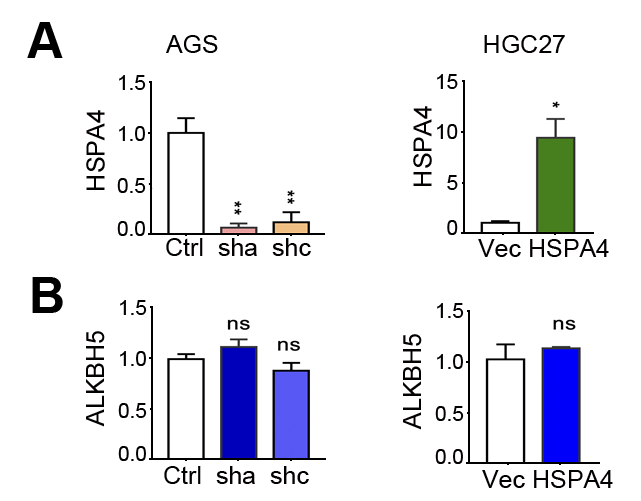
**

**Supplementary figure 1: The RNA levels of HSPA4 and ALKBH5 in GC cells.**

**A**, **B**) The RNA levels of HSPA4 (**A**) and ALKBH5 (**B**) in GC cells with HSPA4 knockdown or overexpression. (*, *P*<0.05; **, *P*<0.01; ns, nonsignificant)


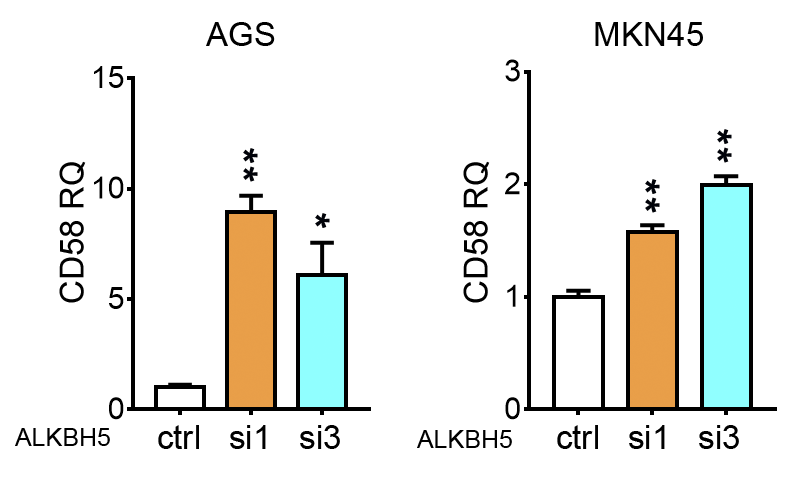


**Supplementary figure 2: The RNA level of CD58 in ALKBH5 knockdown GC cells.**

The RNA level of CD58 in ALKBH5 knockdown GC cells was analyzed q-PCR (*, *P*<0.05; **, *P*<0.01).

**
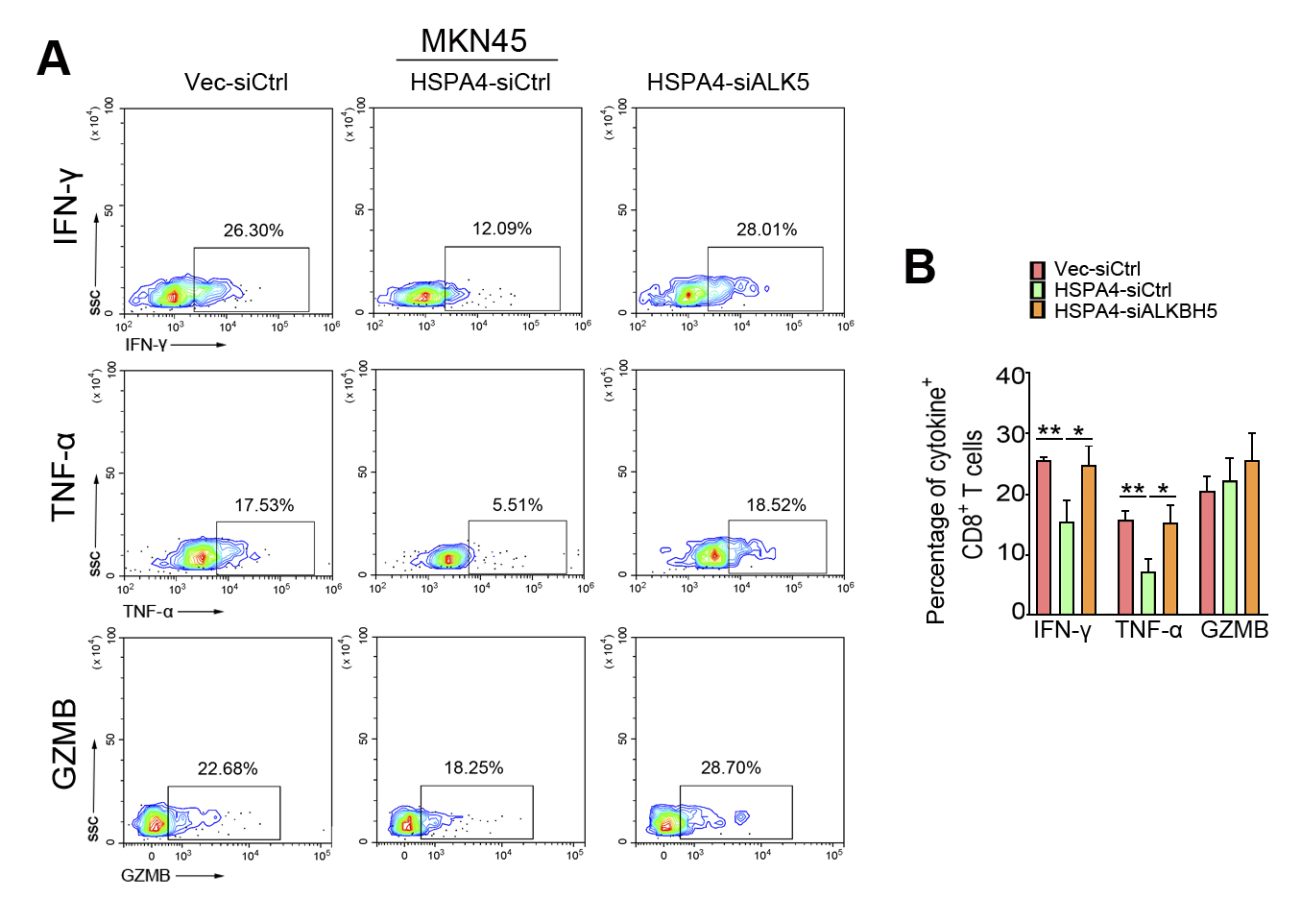
**

**Supplementary figure 3: FACS analysis of CD8^+^ T cells co-cultured with HSPA4 and ALKBH5 manipulated MKN45 cells.**

CD8^+^ T cells were co-cultured with MKN45 cells with HSPA4 overexpression and silencing ALKBH5 for 16 h. CD8^+^ T cells were then stained with IFN-ɤ, TNF-α, and GZMB followed by flow cytometry. Representative pictures and summary of IFN-ɤ, TNF-α, and GZMB positive CD8^+^ T cells (*, *P*<0.05; **, *P*<0.01).

**Quantification of western blotting results:**


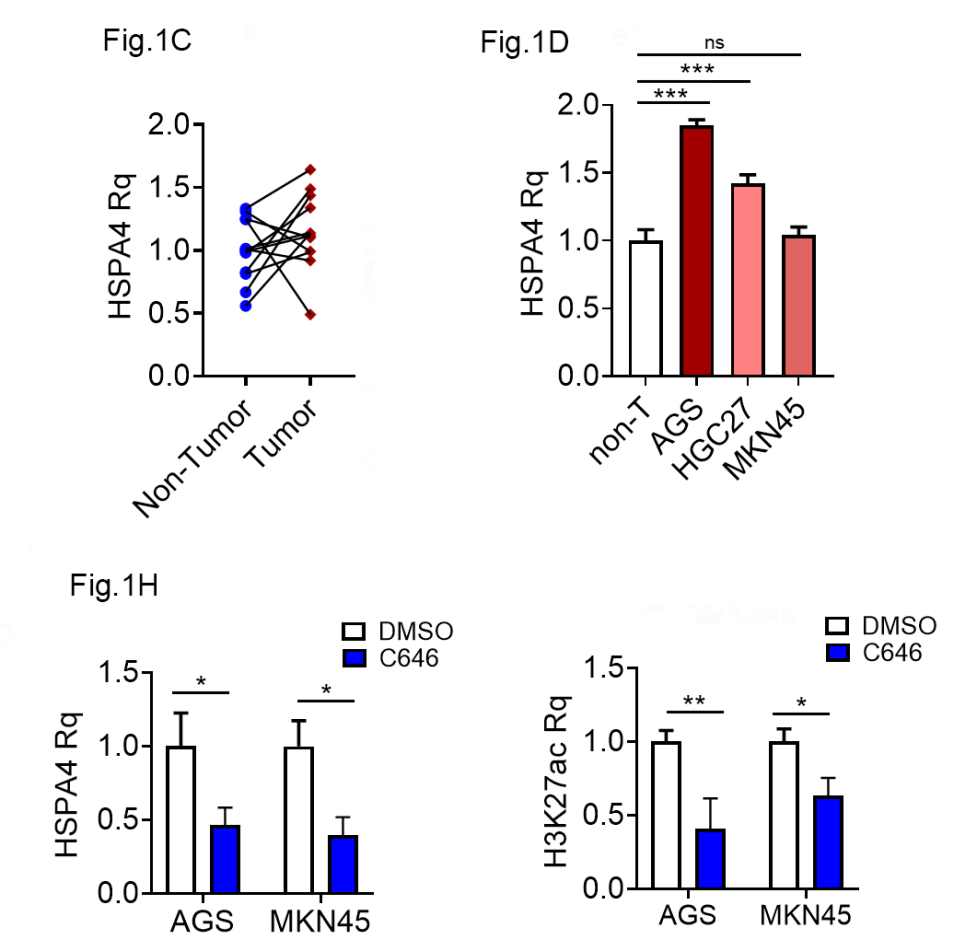


**Quantification of western blotting results in Figure 1.**

Quantification of western blotting images were performed using Image J. (*, *P*<0.05; **, *P*<0.01; ***, *P*<0.001; ns, not significant)


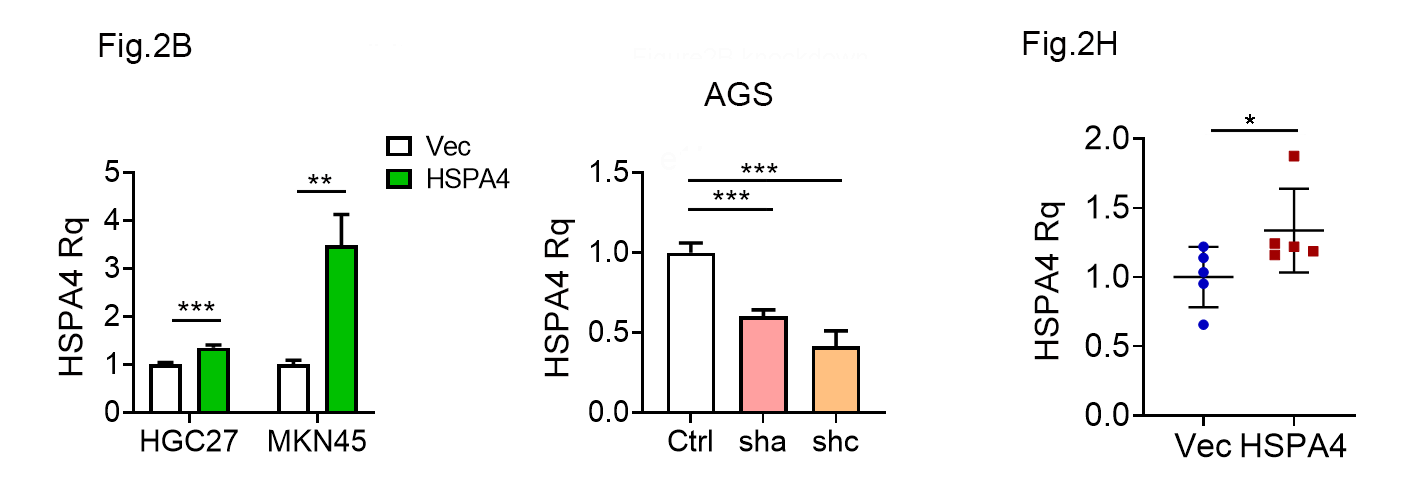


**Quantification of western blotting results in Figure 2.**

Quantification of western blotting images were performed using Image J. (*, *P*<0.05; **, *P*<0.01; ***, *P*<0.001)

**
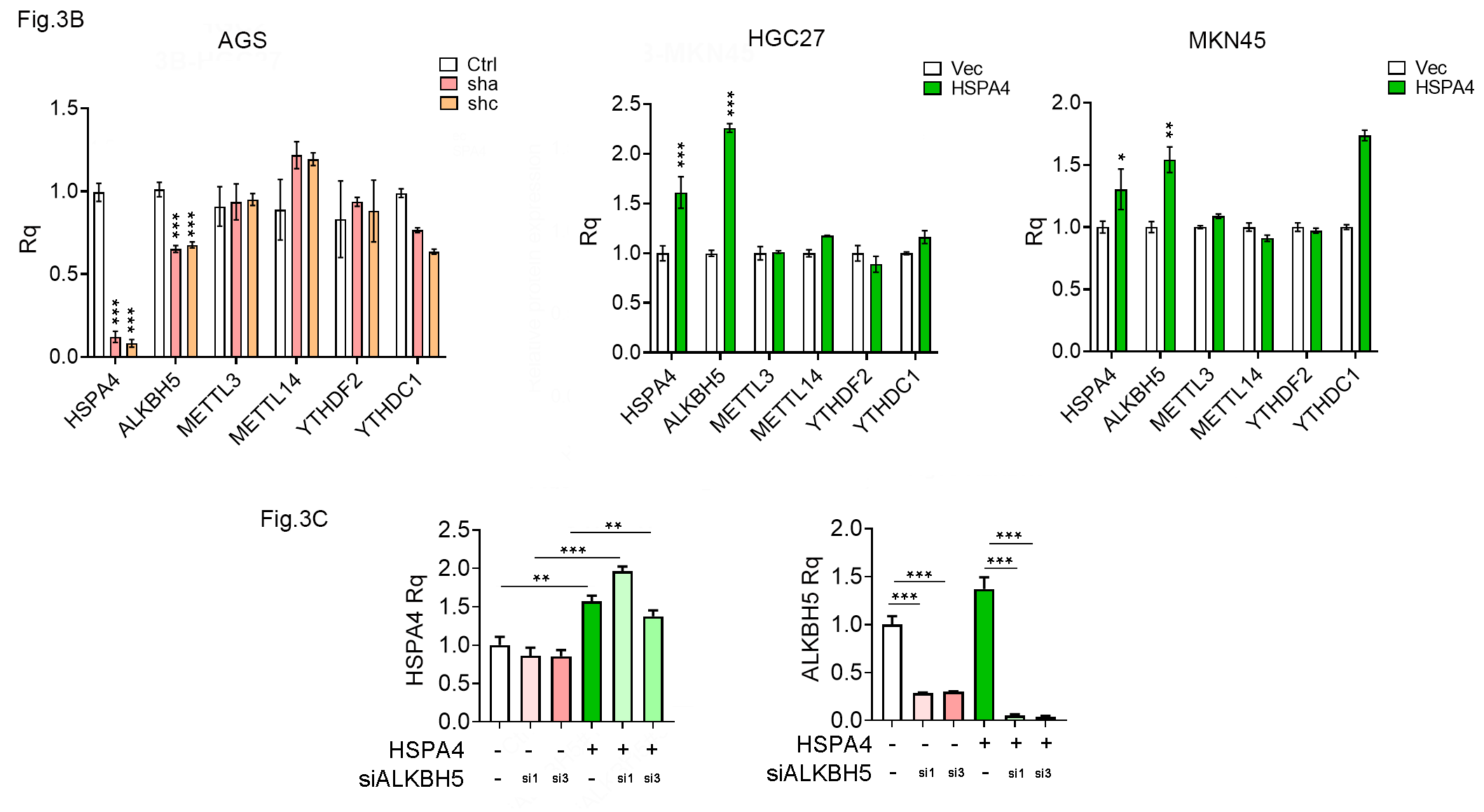
Quantification of western blotting results in Figure 3.**

Quantification of western blotting images were performed using Image J. (*, *P*<0.05; **, *P*<0.01; ***, *P*<0.001)

**
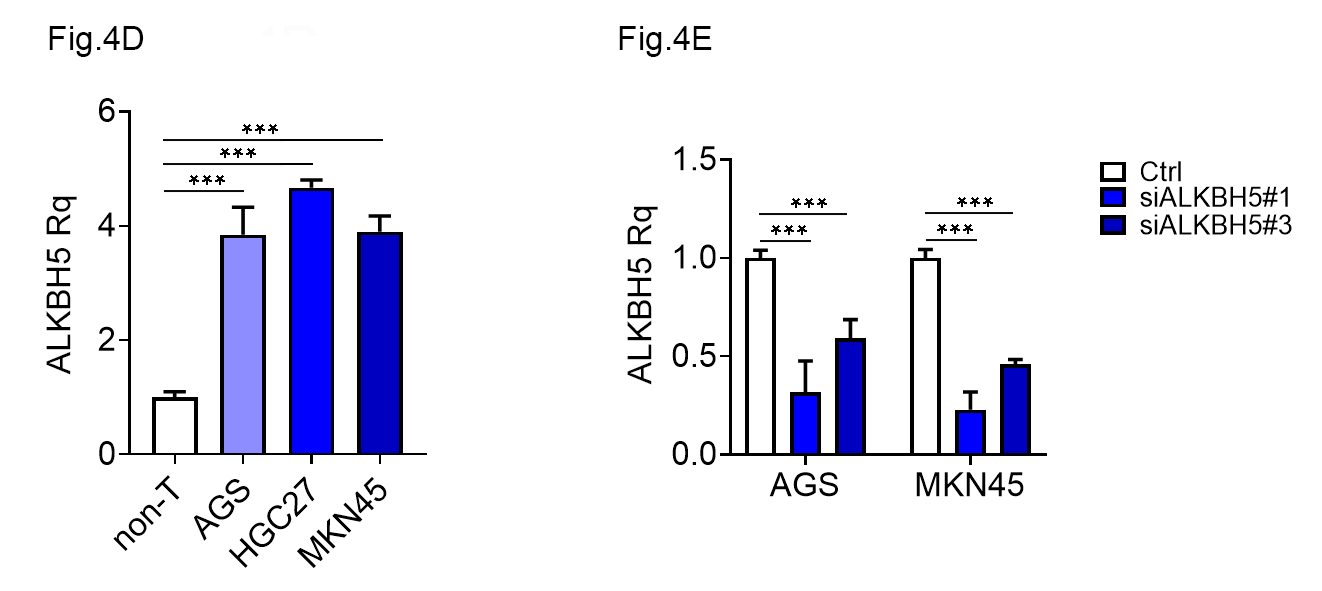
**

**Quantification of western blotting results in Figure 4.**

Quantification of western blotting images were performed using Image J. (***, *P*<0.001)
